# Human anti-thrombospondin type 1 domain-containing 7A antibodies induce membranous nephropathy through activation of lectin complement pathway

**DOI:** 10.1042/BSR20180131

**Published:** 2018-06-21

**Authors:** Zheng Wang, Lu Wen, Yanna Dou, Zhanzheng Zhao

**Affiliations:** 1 Department of Nephrology, Nephrology Hospital of The First Affiliated Hospital of Zhengzhou University, Zhengzhou 450052, China; 2 Institute of Nephrology, Zhengzhou University Institute of Nephrology, Zhengzhou, China

**Keywords:** Anti-THSD7Aantibodies, lectin complement pathway, Mannose-Binding Lectin, membranous nephropathy

## Abstract

To investigate whether the human anti-thrombospondin type 1 domain-containing 7A (THSD7A) antibody-induced membranous nephropathy (MN) is mediated by activating lectin complement pathway. Automatic biochemical apparatus was used to assess renal function of mice. The serum levels of anti-THSD7A antibodies and complement were tested by using ELISA. The expression level of THSD7A and mannose-binding lectin (MBL) in clinical tissue, and the histological features of MN in mice were examined by immunochemical methods. We found that THSD7A, MBL, and complement expression level from patients with circulating anti-THSD7A antibodies were significantly higher than that in normal group. Furthermore, difference of renal function in anti-THSD7A antibody-containing serum treatment groups and control groups was significant. Meanwhile, human anti-THSD7A autoantibodies activated the complement system and induced the histological features of MN in mice. In conclusion, human anti-THSD7A antibodies induce MN through activating MBL lectin complement pathway in mice.

## Introduction

Membranous nephropathy (MN) is a common cause of nephrotic syndrome. It is characterized by the presence of immune complex deposits along with the glomerular basement membrane (GBM), resulting in thickening of the GBM and podocyte foot process effacement [1]. MN was divided into idiopathic MN (IMN) without identified causes and secondary MN (SMN) caused by various autoimmune diseases, infections, and cancers [2]. Of note, the number of patients with IMN accounted for ~70% of patients with MN [3]. Some researches pointed out that the specific antibodies can recognize the cell antigen and activate complement, which plays an important role in the pathogenesis of IMN [4]. The autoantibodies in IMN can recognize and bind to the target antigens on glomerular podocytes and eventually formed immune complexes glomerular capillary walls [5]. In addition, research suggests that membrane attack complex (MAC) activates the complement system at the basement membrane zone [6] to lead to proteinuria and hypoalbuminemia [7].

The activation of complements is involved in classical pathway, alternative pathway, and the lectin pathway. Amongst them, the lectin pathway is activated through the mannan combined with lectin (mannose-binding lectin, MBL) to identify glycoprotein on bacterial surface [8]. The research indicates that the MBL complement pathway induces IgA nephropathy with the glomerular lesions [9]. Intriguingly, IgG4 is known to be effective to trigger the MBL pathway activation in human IMN [10].

M-type phospholipase A2 receptor (M-PLA2R), a subtype of IgG4, was identified as a specific pathogenic target antigen in most patients with IMN [11]. It has been reported that expression of MBL-associated serine protease-1 (MASP-1), MASP-2, and MBL is a significantly elevated in the patients with positive PLA2R antibodies, indicating that anti-M-PLA2R could activate the lectin complement pathway [10,12]. Several studies pointed out that anti-PLA2R is predominating in the kidneys of patients with IMN, and have the potential to become biomarker of the differential diagnosis of IMN and monitor the prognosis of disease [10,13].

The human anti-thrombospondin type 1 domain-containing 7A (THSD7A) antibodies are also a subclass of the IgG4. The structure and biochemical properties of THSD7A are similar to PLA2R1 [14]. In recent years, some scholars have found that the THSD7A existed in IMN patients with negative PLA2R [15–17]. This provides evidence to support that THSD7A antibodies indeed cause MN. However, the underlying mechanisms of the anti-THSD7A antibodies inducing the MN are still unclear. In our research, we investigated whether anti-THSD7A antibodies could cause MN in mice by activation of lectin complement pathway.

## Materials and methods

### Clinical specimens

Participants included 25 patients with IMN, 13 patients with renal cell carcinoma, and 18 healthy volunteers (all aged ≥18 years, male or female). All patients were treated without hormone and immune inhibitor. The renal biopsy tissue screened was from IMN and normal renal cortical tissue of patients undergoing nephrectomy for renal cell carcinoma (as control). Blood samples were obtained from patients with IMN and healthy controls and anti-THSD7A antibodies in serum were examined using ELISA. All study participants gave written informed consents, and the study was approved by the Ethics Committee of The First Affiliated Hospital of Zhengzhou University.

### Experimental animals

Twelve to fourteen weeks old BALB/c male mice were purchased from Beijing HFK Bioscience Co., Ltd. (Beijing, China), weighing 22–30 g. All experimental protocols were approved by the Institutional Animal Care and Use Committee of The First Affiliated Hospital of Zhengzhou University. The animals had free access to water and standard animal chow. The anti-THSD7A antibody-positive serum from patients with THSD7A-associated IMN was used for disease induction, serum was from healthy donor, and saline solution served as a negative control. The mice were randomly divided into three groups and the treatments were as follows: (i) control group (*n*=10), in which mice were injected intraperitoneally with 900 μl saline solution; (ii) normal serum group (*n*=10), in which mice were injected intraperitoneally with 900 μl serum of healthy volunteers; (iii) model group (*n*=10), in which mice were injected intraperitoneally with 900 μl anti-THSD7A antibody-containing sera from patients with IMN. In anti-THSD7A antibody group, 24 h urine protein excretion <5 mg was not selected into the model group after 1 week of injection. Two, four, and seven weeks after the treatment, urine samples were collected in each group. Blood was taken from the heart and kidneys of anesthetized animals, for analysis 7 weeks after treatment.

### Histopathological examination immunostaining

Paraffin sections (3 μm) of renal tissue from the patients with IMN and normal renal cortical tissue 7 weeks after the treatment (described above) were deparaffinized, rehydrated, and repaired, then stained using Hematoxylin and Eosin (H&E), Periodic Acid-Silver Metheramine (PASM), and Masson’s staining (all from Sigma-Aldrich) according to manufacturer’s guidelines or the sections were dewaxed, rehydrated, and repaired. Endogenous peroxidase activity was quenched using 3% hydrogen peroxide in methanol for 10 min. Then the sections were stained with rabbit anti-THSD7A (1:400, Atlas), anti-nephrin (1:200, Boster, Wuhan, China), anti-MBL (1:200, Union-Biotechnology), anti-C3b (1:200, Shanghai Kanglang Biological Technology Co., Ltd., Shanghai, China), anti-C5b-9 (1:500, Abcam, Cambridge, MA) at 4°C overnight, then washed twice with PBS and incubated with the corresponding secondary antibody for 30 min at 37°C. 3,3′-Diaminobenzidine (DAB, Vector Laboratories) was used as the chromogenic substrate according to the manufacturer’s instructions. Finally, sections were counterstained using 10% Hematoxylin (ProSciTech). Histology pictures were captured using a microscope with a digital camera (Nikon, Japan) and analyzed by ImagePro Plus 6.0.

### ELISA analysis

We collected the venous blood of healthy volunteers and IMN patients to separate serum. Serum levels of anti-THSD7A antibody and complement were evaluated using anti-THSD7A, MASP-1, MASP-2, MBL, C3a, C5a, and sC5b-9 ELISA kits in accordance with the manufacturer’s instructions. All ELISA kits were purchased from Huamei Biological Engineering Co. Ltd. (Wuhan, China).

### Biochemical assay

Level of the total protein (TP), albumin (ALB), cholesterol (CHOL), triglyceride (TG), and serum creatinine (Scr) in the urine samples and serum was evaluated by using a fully automatic biochemical apparatus (Beckman CX7; Beckman Coulter, CA, U.S.A.).

### Statistical methods

The data are expressed as the mean ± S.D. All statistical analyses were done using SPSS 13.0 software (IBM SPSS, Armonk, NY, U.S.A.). Statistical significance was determined using Student’s *t*test or one-way ANOVA followed by *SNK* test. *P*<0.05 was considered to be statistically significant.

## Results

### The expression of THSD7A and complement ingredients

**Figure 1:**
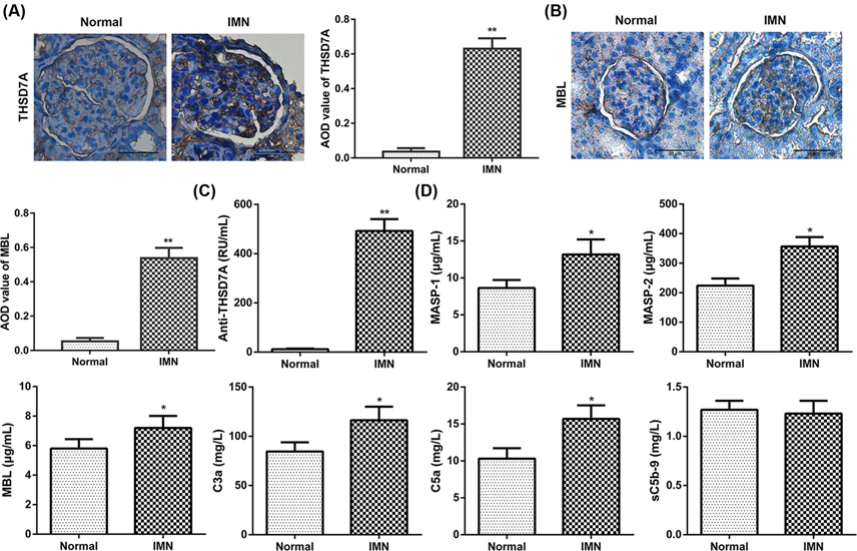
Expression of THSD7A and complement protein in IMN (**A**) The expression of THSD7A in different groups. (**B**) The expression of MBL in different groups. Scale bars = 20 μm. (**C**) The expression of serum anti-THSD7A antibody in different groups. (**D**) The expression of MASP-1, MASP-2, C3a, C5a, sC5b-9 in different groups. *P<0.05 compared with normal group, **P<0.01 compared with normal group.

We first screened renal biopsy tissue from patients with IMN, and normal renal cortical tissue from patients with renal cell carcinoma undergoing nephrectomy as a normal control (Normal). Expression of THSD7A and MBL in IMN and normal groups was detected using immunohistochemical staining. The results showed that staining of THSD7A and MBL was notably stronger in the IMN group than than in the normal group. (Figure 1A,B).

Furthermore, the ELISA was performed to analyze serum levels of anti-THSD7A antibodies and lectin complement ingredients (MASP-1/2, MBL, C3a, C5a, and sC5b-9) in each group. As shown in Figure 1C,D, levels of anti-THSD7A antibodies and MASP-1/2, MBL, C3a, C5a in serum were markedly increased in IMN group compared with that in normal group. However, there were no statistically significant differences in sC5b-9 level between the two groups. All the results indicate that complement system proteins are activated in patients with positive anti-THSD7A antibodies.

### Anti-THSD7A autoantibodies of patient with MN activate complement in mice

**Figure 2:**
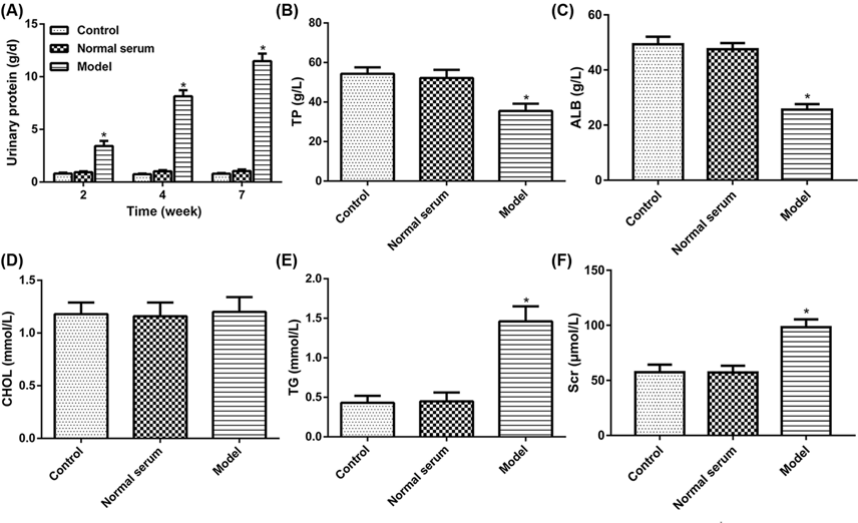
Anti-THSD7A antibodies impair renal function in mice (**A**) Assessment of 24 h urine protein contents. (**B**) Assessment of TP content. (**C**) Assessment of ALB content. (**D**) Assessment of CHOL. (**E**) Assessment of TG. (**F**) Assessment of the Scr. *P<0.05.

**Figure 3:**
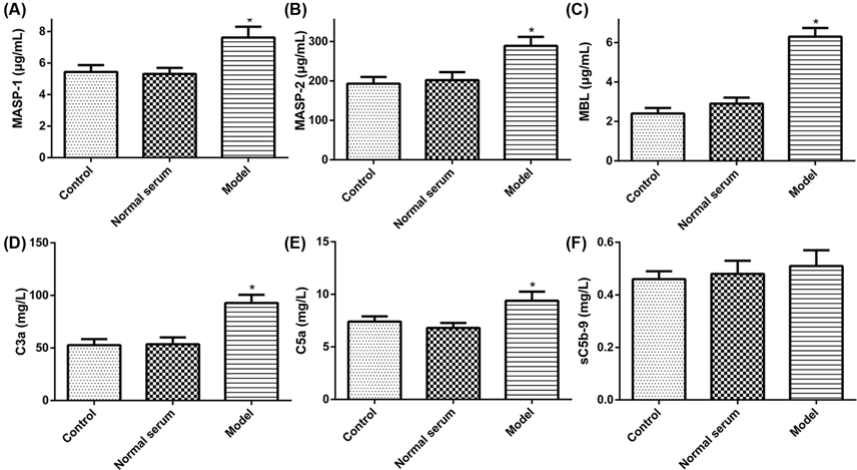
Serum complements levels in different experimental groups (**A**) The content of MASP-1. (**B**) The content of MASP-2. (**C**) The content of MBL. (**D**) The content of C3a. (**E**) The content of C5a. (**F**) The content of sC5b-9. *P<0.05.

Based on the results obtained from the renal biopsy tissue and serum of IMN, we next examined whether the injection of human anti-THSD7A antibody in serum causes complement activation in mice. First, 24 h urine protein excretion of mice after injecting for 2, 4, and 7 weeks were measured by using automatic biochemistry analyzer. The results showed that model group in 24 h urine protein content was significantly increased at all time points than control group, while the levels of the 24 h urine protein in normal serum group had no obvious change (Figure 2A). Meanwhile, blood biochemical determination showed that level of TP and ALB in the model group was markedly reduced compared with that in the normal group, but the content of TG and Scr was significantly increased, and the CHOL level had not obviously changed (Figure 2B–F). Besides, we obtained serum samples from mice injected with human anti-THSD7A antibody containing serum or normal serum or saline solution up to 7 weeks. The results showed that serum MASP-1, MASP-2, MBL, C3a, C5a, and sC5b-9 levels were tested in mice by ELISA. The results showed that the expression of MASP-1, MASP-2, MBL, C3a, and C5a in the model group had a clear rising as compared with that in normal group, but the expression of sC5b-9 had no distinct change (Figure 3). These data implied that the anti-THSD7A antibodies might influence lectin complement pathway.

### Human anti-THSD7A antibodies induce the histological features of MN in mice

**Figure 4:**
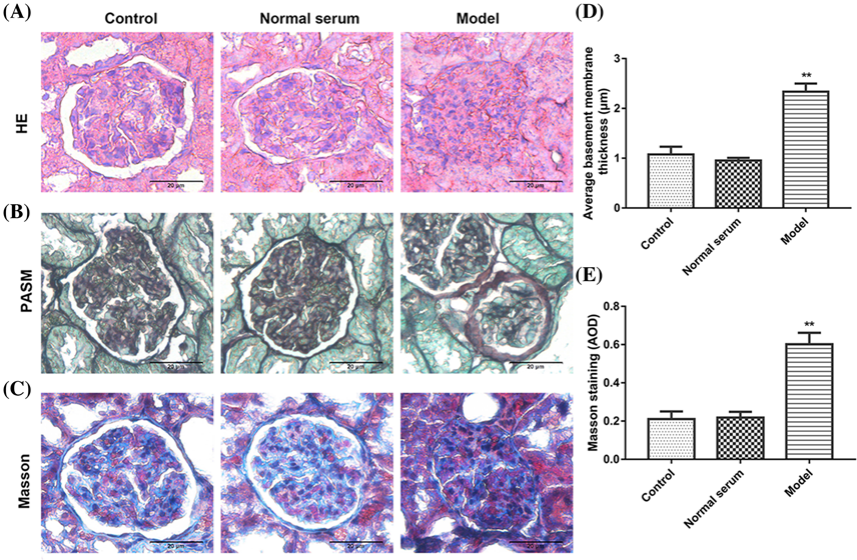
The pathological changes in renal tissues in mice (**A**) HE, (**B**) PASM, (**C**) Masson’s staining. (**D**) Quantitative analysis of PASM staining. (**E**) Quantitative analysis of Masson’s staining. Scale bars = 20 μm. **P<0.01 compared with control.

Given the above-described clinical data, we observed that anti-THSD7A antibodies and lectin pathway mediated complement levels were up-regulated in IMN, and the human anti-THSD7A antibodies actived lectin complement pathway in mice. We hypothesized that human anti-THSD7A antibodies might cause MN in mice. To investigate the pathogenicity of human anti-THSD7A antibodies in mice, pathological change in renal tissues from mice injected with anti-THSD7A antibody containing serum from patients with MN was observed by immunostaining. As is shown in Figure 4, histological stain (HE, PASM, and Masson) of model group had obvious changes in glomerular structures, mesangial cells hyperplasia, the thickness of the GBM, the glomerular volume became bigger, and visible balloon adhesions, but had no obvious changes in tissue structure in normal serum group and control group (Figure 4A–C).

**Figure 5:**
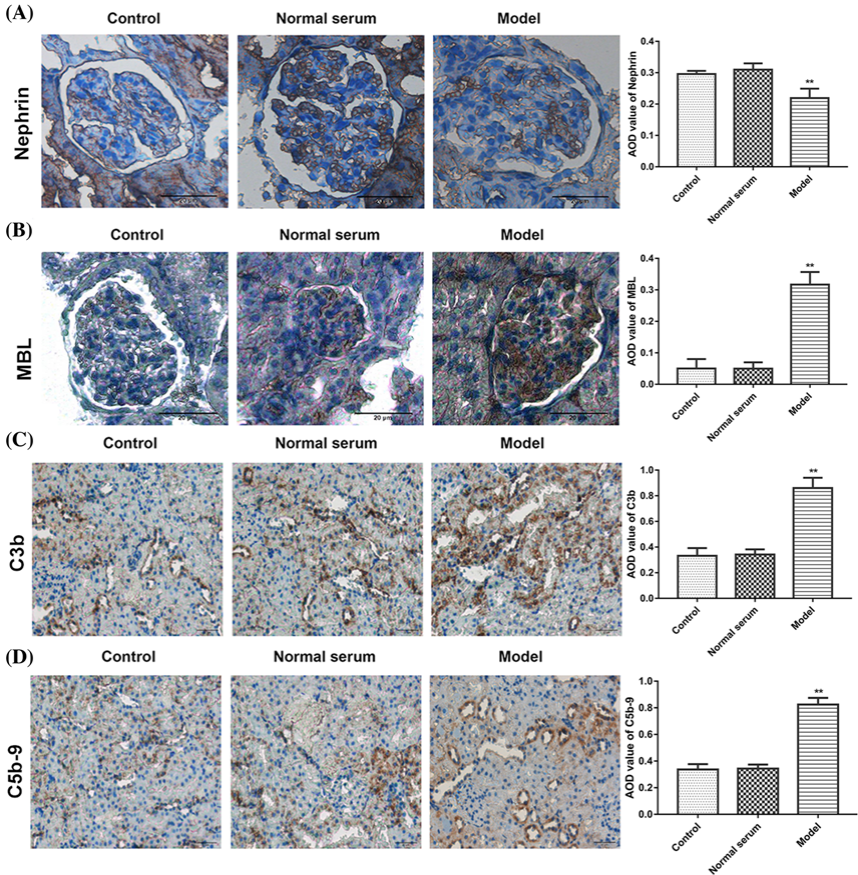
Level of nephrin, MBL, C3b, and C5b-9 expression in renal tissues of mice (**A**) Immunohistochemical analysis of nephrin. (**B**) Immunohistochemical analysis of MBL. (**C**) Immunohistochemical analysis of C3b. (**D**) immunohistochemical analysis of C5b-9**.** Scale bars = 20 μm. **P<0.01 compared with control.

In addition, immunohistochemistry of renal tissues in normal serum group and control group showed that the distribution of nephrin was localized along the GBM in a finely granular or linear pattern (brown). However, compared with control and normal serum groups, a weaker, sparser, and diffused interrupted linear pattern of nephrin expression was observed in the model group (Figure 5A). Meanwhile, MBL, C3b, and C5b-9 staining remarkably increased in model group than that in the control and normal group (Figure 5B–D). These data suggested that anti-THSD7A antibodies induce activation of lectin complement pathway and MN in mice.

## Discussion

MN is the most common autoimmune disease amongst adults, with up to ~60–80% of the patients with IMN [18,19]. IMN is an antibody-mediated autoimmune disease. Pathogenicity antigen of glomerular podocytes causes formation of immune complex *in situ* and activation of the complement system, leading to a change in the structure of the basement membrane, filtration barrier, and proteinuria [7]. The research indicates that human MN is associated with the discoveries of neutral endopeptidase (NEP) PLA2R1 and THSD7A [20–22].

PLA2R1 is expressed on the basal surface of glomerular podocytes and serves as the major antigen involved in the pathogenesis of IMN [11]; and ~70–80% of patients with IMN have circulating autoantibodies against PLA2R1 [23,24]. A recent study suggests that THSD7A is a novel MN-causing antigen and estimates to underlie 5–10% of cases of IMN in patients with serum negative for anti-PLA2R1 antibodies [14,17]. In our research, expression of THSD7A and MBL in IMN showed an enhanced staining than those in control group. In addition, lectin complement proteins were markedly increased in IMN group compared with that in normal group. These studies indicated that anti-THSD7A antibodies and complement system proteins are activated in patients with IMN.

The anti-THSD7A and PLA2R1 serum antibodies are predominantly of the IgG4 subclass by recognizing the corresponding target antigen to start a series of changes in IMN [22,25,26]. It has been reported that patients with continuous IgG4 positivity are able to activate lectin complement pathway in IMN [10,27]. Accordingly, previous research found that PLA2R antibody could activate complement-lectin pathway [28]. Complement, an important component of the innate immune system, plays an important role in host defense reaction [29]. In the present study, we found that human anti-THSD7A antibodies promoted serum MASP-1, MASP-2, MBL, C3a, C5a expression, reflecting the anti-THSD7A antibodies involved in lectin complement pathway in mice.

The damage of podocyte is the important factor to lead the pathology of glomerular tissue [30]. THSD7A has been proved to express in podocyte, which cause the development of MN [31]. In our experiments, histological staining of tissues from mice treated with anti-THSD7A antibody had an obvious change in glomerular structures, mesangial cells hyperplasia, the thickness of the GBM, the glomerular volume became bigger, and visible balloon adhesion. Besides, immunohistochemistry of renal tissues in normal serum group and the control group showed that the distribution of nephrin was localized along the GBM in a finely granular or linear pattern. Compared with control and normal serum groups, the model group had a weaker, sparser, and diffused interrupted linear pattern of nephrin expression. Furthermore, MBL, C3b, and C5b-9 staining in model group was remarkably increased than that in control and normal groups. These results revealed that anti-THSD7A antibodies induce activation of lectin complement pathway and pathological process of IMN in mice.

In conclusion, our study demonstrates that human anti-THSD7A antibodies induce the IMN by local activation of the complement system in mice. This finding not only further helps to elucidate the pathogenesis of IMN but also allows for the potential identification and monitoring of patients with serum positive for anti-THSD7A autoantibodies.

## Abbreviations

ALB: albumin
CHOL: cholesterol
GBM: glomerular basement membrane
HE: heamatoxilin–eosin
IMN: idiopathic membranous nephropathy
MASP: mannose-binding lectin associated serine protease
MBL: mannose-binding lectin
MN: membranous nephropathy
M-PLA2R: M-type phospholipase A2 receptor
PASM: Periodic Acid-Silver Metheramine
PLA2R: phospholipase A2 receptor
Scr: serum creatinine
TG: triglyceride
THSD7A: thrombospondin type 1 domain-containing 7A
TP: total protein
SNK: Student -Newman-Keuls
sC5b-9: soluble terminal complement complex
